# Experiments in interdisciplinarity: Responsible research and innovation and the public good

**DOI:** 10.1371/journal.pbio.2003921

**Published:** 2018-03-26

**Authors:** Ana Delgado, Heidrun Åm

**Affiliations:** 1 TIK Centre for Technology, Innovation and Culture, University of Oslo, Oslo, Norway; 2 Department of Interdisciplinary Studies of Culture, Norwegian University of Science and Technology, Trondheim, Norway; City University of London, United Kingdom of Great Britain and Northern Ireland

## Abstract

In Europe, responsible research and innovation (RRI) has emerged as a science policy measure that demands the early integration of a broad range of social actors and perspectives into research and development (R&D). More collaboration of the social sciences and humanities (SSH) with science and engineering appears within this policy framework as a crucial element that will enable better technological development. However, RRI is new to both natural scientists and SSH scholars, and interdisciplinary collaborations are challenging for many reasons. In this paper, we discuss these challenges while suggesting that what RRI can be in a particular project is not a given but remains an empirical question. Natural scientists and SSH scholars need to coresearch RRI in an experimental mode.

This Perspective is part of the *Public Engagement in Science* Series.

## Responsible research and innovation and the public good

In Europe and beyond [1,2,3], the science policy idea of responsible research and innovation (RRI) has gained momentum in both national and supranational funding agencies and institutions. Broadly, policy documents often present RRI as an attempt to enhance science governance in the pursuit of better research and innovation for the public good. Key to its science policy, the European Commission (EC) has identified a number of significant “societal challenges” and requires researchers to design their projects to address such challenges. The EC implemented RRI in three ways in the European Union’s Horizon 2020 program: by funding RRI research as well as coordination and support projects; as actions to implement RRI in universities and other institutions; and as a crosscutting issue in funding programs and research projects [4]. RRI builds on previous developments in science policy that revealed the need to engage with sectors of the public in decisions about science technology as early as possible in the scientific research [5,6]. RRI demands integrating a broad range of stakeholders into research and innovation projects to address societal challenges for which these actors share responsibility [7]. Interdisciplinarity is also a locus of such policy demands for integration. In particular, the Vilnius Declaration from 2013 expressed concern about how the social sciences and humanities (SSH) can be incorporated in Horizon 2020 [8]. The collaboration between science and engineering and SSH is key in RRI [9,10]; such collaboration shall enable the production of research and innovation “with and for” society [11].

In national contexts, EU projects play an important role in the adoption of RRI. Beyond EC funding schemes, national funding agencies in countries such as Norway, the United Kingdom, and the Netherlands promote the integration of RRI in research projects. The Research Council of Norway, for instance, requires that researchers applying to biotechnology, nanotechnology, and Information Communication Technology (ICT) funding programs describe in their proposals how they plan to conduct their research according to RRI frameworks. In the UK and Norway, RRI frameworks highlight the dimensions of inclusion, anticipation, reflection, and responsiveness to inform research and innovation practices [12,13]. Definitions of RRI are diverse [14,15]. Yet arguably, components for realizing RRI in research practices—such as public engagement, interest in value pluralism, and interdisciplinary collaborations [16]—coincide with a concern for the production of science for the “public good.” And even though RRI requires that research and innovation are oriented toward the public good, what the “public good” entails in particular contexts remains undefined. If research and development (R&D) actors, for instance, interpret the public good mainly in terms of market needs, that would not counter tendencies to depoliticize science [17]. Furthermore, research calls and programs preclude defining how R&D actors shall translate RRI into practice. Rip [18] points out a lack of definition as a general characteristic of RRI policies.

In our view, such definitions should occur within the context of specific interdisciplinary collaborations and remain an empirical question. A lack of definition could lead to misunderstandings that could undermine such collaborations. However, it can also provide room for inventiveness, which is a necessary condition for experimentation. In this paper, we explore these issues by first identifying three key challenges RRI poses in interdisciplinary collaborations, drawing on our experiences as social scientists working with natural scientists, primarily in the life sciences in Norway: newness, indeterminacy, and complexity. Second, we invite the reader to think of these challenges not as limiting attributes of RRI but as experimental conditions.

The concept of the experiment that we use here emphasizes the open-ended nature [19] and productive potentials of this practice [20]. RRI can certainly be seen as an experimental approach to science governance, in the sense that it demands that research be oriented toward producing a certain result (i.e., better technological futures or the public good). But the means to reach the goal must be set up under conditions of uncertainty, as in experimental designs. The importance of experimentation as a dimension of inclusive and collaborative research processes [21], and in particular RRI, has been pointed out [13]. In this paper, we align with and advance these views by focusing on interdisciplinary collaborations in RRI as experiments. What RRI in a particular project is appears in this light as an empirical question, which needs to be researched by the various project participants.

## Challenges in RRI interdisciplinary collaborations: Newness, complexity, and indeterminacy

### Coping with a new situation, moving out of the comfort zone

RRI is a relatively new policy measure that, since 2011, has caught on within the structures of the EC [22]. In Norway, as in other places, the emergence of RRI took most natural scientists by surprise. Principal investigators (PIs) in the natural sciences first encountered RRI as a mandatory requirement when writing a project proposal for a funding call (in which SSH scholars would typically be invited to participate, being rarely able to apply to funding schemes of this sort as project leaders). Therefore, PIs in the natural sciences faced RRI in the context of a sudden and urgent need to find someone who “does RRI” before the call deadline. In these situations, scientists in biotechnology and related fields have approached us on a number of occasions to contribute to their project proposals. However, it is difficult to be constructive partners in projects that pursue research questions and objectives that we were unable to shape from an SSH perspective. Some SSH scholars may even deem such last-minute calls as inappropriate, and most undoubtedly struggle with the demand to produce a quick solution under a tight deadline. Because RRI is new to both natural scientists and SSH scholars, policy demands for increased collaboration challenge the usual practices in both fields. For instance, PIs in the natural sciences tend to turn to stakeholder workshops as a quick solution to deal with RRI. SSH scholars can see such workshops as providing a service or as supplementing the knowledge produced by natural scientists without actually merging with it. However, as social scientists, we are not necessarily comfortable when suddenly assigned tasks (e.g., organizing stakeholder workshops) that are outside our core activity (i.e., doing research) or know-how. In particular, we might not share life scientists’ urgency to produce a faster uptake of scientific results and public acceptance of technology. Instead, SSH scholars may see value in public debate as a way of opening technological trajectories up for discussion and as a way to arrive at enriched definitions of research issues and problems [23].

Similarly, RRI may present an uncomfortable experience for natural scientists who must suddenly accommodate their project designs to new and unfamiliar requirements. For instance, research funders might encourage them to welcome social scientists into their labs or to allocate money and time for stakeholder workshops without actually explaining to the natural scientists why this is necessary or helpful to make their research better. Furthermore, funding programs increasingly require natural scientists to show how their research directly addresses grand societal challenges, but doing so might not always be easy. Therefore, RRI takes both natural science and SSH scholars out of their comfort zones, pushing them into new situations and confronting them with new sorts of problems. Answers to such problems are not forthcoming but instead need to be codeveloped on the empirical specificities of each research project. The next section points to several such empirical specificities.

### Complexity in science–society relations

SSH scholars have reported on how RRI translates into scientific practices [24] and on how natural scientists may experience this demand as an added difficulty. Science is naturally changing, and scientists are under increasing pressure to publish, write applications, engage in international mobility programs, and to deliver fast. Time certainly structures research [16], and natural scientists can encounter RRI requirements as obstacles to doing “good science” [25]. Yet, we argue, “good science” is not a given.

Although RRI requests that innovation-oriented science and technology be aligned with social needs and values, we have been invited to take part in project applications that propose rather technical solutions to scientific problems, failing to address real-world problems in their intricacy. If the distinction between basic and applied science was always blurred [26], this may be even more the case as research is increasingly innovation driven. In practice, scientists may experience difficulties adjusting their research interests, background, or trajectories to what funders deem more socially relevant territories. In addition, current demands for producing more of what is called socially relevant science coexist with an institutional push for scientific excellence. This creates tensions and challenges to finding the right balance when designing scientific projects. Furthermore, new demands for open science also influence scientific practices (from publishing to data storage). At stake within these demands is a redefinition of what constitutes good science.

Understanding the intricate relations between science and society and their manifold possible tensions is a demanding task, and SSH scholars have been developing conceptual tools for this purpose. Importantly, RRI demands that the focus of the relation between different public groups and scientists be shifted from producing acceptable results to producing responsible innovation [27]. This entails new and more inclusive forms of technological interventions. Indeed, the first “R” in RRI, “responsible,” refers to a kind of research that can respond to and integrate public concerns from the very beginning. However, in what concrete ways should public concerns be included? Exploration of public concerns needs to be theoretically informed [28,29,30], which requires SSH literacy. For instance, we might take as a point of departure that technological developments affect people’s everyday lives, sometimes in unexpected ways, and not only in terms of risk. Moreover, issues of public concern may not necessarily overlap with the grand societal challenges identified by institutions, and perhaps they are not in accordance with demands for innovations that create market revenues.

### Indeterminacy and the making of better technoscientific futures

RRI as a policy remains nebulous partly because its results are codified in undefined terms (e.g., the “public good”) and because there is no agreement on how to reach such results. The research tools that RRI practitioners have produced since 2015 have helped systematize RRI to a limited extent [31], but these may also have added to misunderstandings and confusion. For instance, while developing a project proposal, a PI asked one of us whether RRI is research or just something to be implemented. We would answer that RRI is not box ticking. Furthermore, standardizing this way of performing research is neither desirable [32] nor probably even possible.

Crucially, as a policy framework, RRI is oriented toward enabling better futures (with a focus on anticipation). However, when writing project applications, PIs in the life sciences may find it difficult to include such future-oriented focus in their proposals, particularly when technological applications appear to be unrealistic. Promising astounding results can be seen as a form of irresponsible representation [33] of what the technology can actually offer. This is because each scientific project has a particular take on innovation, which in turn requires specific forms of RRI research. Whereas the general goal is producing research for the public good, the particular ways in which this might happen requires its own research, which would be best set in an experimental and collaborative mode. RRI is experimental in the sense that it requires novel types of collaborations, the shape of which is undetermined and the results of which are uncertain [34]. Arguably, RRI collaborations will enable better technological trajectories, but technological trajectories are such experimental objects: they can be designed and steered, but their outcomes cannot be predicted.

## From challenges to experimental conditions: RRI as interdisciplinary collaborations

RRI challenges all the involved parties into producing knowledge in new ways. As we have outlined, RRI demands from all involved parties that they collaborate in a research situation that is to a large extent unknown and undefined, which can lead to misunderstandings and confusion. At the same time, the success of such collaboration depends on acknowledging and engaging with the complexity of science–society relations. When we attend to those complexities, each research project reveals itself to have a unique setting, a specific empirical domain.

The challenges presented above, however, do not necessarily need to be seen as problems but as exposing the open-ended, empirical, and experimental nature of RRI situations. If the reader has been involved in designing experiments, she’s aware that the experimental situation can lead to new and unexpected questions, and problem-solving—including the creative production of research tools and methods. Here, we apply the inventive and productive nature of experiments [35,36,19,20] to the practice of RRI and provide an example from our own practice. We invite the reader to think of interdisciplinary collaborations as open-ended processes in which novel questions and opportunities for defining and solving problems may arise, thereby needing continuous readjustments.

Between 2011 and 2013, one of us (Ana Delgado) collaborated with researchers at a synthetic biology lab at the University of Valencia (UV) in Spain. At the time, synthetic biology was a relatively new field often presented as having great potential to deal with problems ranging widely from drugs to energy production (biofuels), among others. The UV lab had been doing research on the design of a cell chassis that could be used as a platform for developing such applications. Delgado collaborated in supervising students as they developed their synthetic biology projects for the International Genetically Engineered Machine (iGEM) competition. During this collaboration, “design in synthetic” biology emerged as a theme of research. The PI of the group and Delgado engaged in further research and discussions on this theme, choosing to share them with the synthetic biology community in the form of a research article. In this coauthored paper [37], both the natural scientist and the social scientist problematize design as research practice, particularly in the field of synthetic biology, by critically attending to the assumptions underlying views from the different disciplines involved. The paper also shows how the history, philosophy, and sociology of science can contribute to opening up the notion of “design” as used in synthetic biology, problematizing it but also enriching it. Furthermore, this work led Delgado to question the notion of design not only in synthetic biology but also in her own field—Science and Technology Studies (STS) [38]—and led both authors to further develop a common interest on standardization practices in synthetic biology.

The ultimate aim of this research has been to contribute to better designs and design practices in synthetic biology. What “better” means in concrete terms has remained undefined. Yet that lack of definition is precisely what has provided room for such interdisciplinary discussion while orienting it toward a certain direction. This collaboration was experimental because it attended to the empirical context and conditions of a certain way of doing synthetic biology to open for relevant questions (and questioning). The terms of the collaboration were not settled from the start but were the result of a process of constant adjustment (between the different views) that developed over the course of this relation. The coauthored paper [37] can be seen as a concrete experimental device that allowed both authors to focus (and refocus) their collaboration and to find questions of common interest. This genuine research collaboration challenged the widespread belief that the practice of RRI is to be “outsourced” to SSH scholars, whose role will be to introduce standard versions of the public opinion or to produce public acceptance of the technology at play.

This collaboration exemplifies just one of the many forms that RRI experimental collaborations could take. As an experimental practice, RRI collaborations will need to adjust to particular contexts. Their particular setup, methods, and desired results will need to be coresearched. In each case, the research will need to engage with the intricacies and dynamics of the science–society relations in order to (re)orient them to the production of better common futures. What “better” means here involves both political and scientific considerations that will flow from the specificities of each research project.

## From epistemological conditions to a collaborative ethos

European science policy promotes RRI as a principle for producing research and innovation for the public good, yet incorporating an RRI perspective presents several challenges for natural scientists. We suggest that seeking genuine collaborations with SSH can open a path to engage with such difficulties. Research fields within SSH, such as STS and Technology Assessment, have long been doing research on those tensions and on the intricate relationships between public interest and scientific autonomy; technological uncertainties and regulations; the workings and organization of labs and policies; technological presents and futures; and science and the public—just to mention some long-standing research areas within these fields. These relations are complex and historically configured, and SSH scholarship reveals that they need to be reoriented for the benefit of both science and society. How this will happen remains an open question that requires profound research, experimentation, and learning.

We aspire to unpack and rework those tensions in collaborative settings in order to eventually allow for new and more fruitful science–society relations when doing RRI research and for the sake of collectively imagining better sociotechnical futures and directing action towards achieving them. In this paper, we argue that RRI requires an integration of the natural sciences and SSH disciplines and that the nature of that integration must be researched as an empirical question in accordance with the particularities and needs of each research project. We show that there are challenges in RRI collaborations due to newness, indeterminacy, and complexity. Given these challenges, the question might arise whether it is even wise to implement RRI. We certainly think that RRI is an endeavor that needs to be pursued. That an experiment is challenging to set up should not be sufficient reason to cancel it. Rather, it underscores the importance of approaching RRI as an empirical question requiring coinvestigation.

In this light, humility—in the sense of acknowledging one’s own knowledge limits [39]—appears to be a necessary element of collaboration. Such recognition is not meant to stop us from producing knowledge; instead, it should enable new forms of knowledge production. To approach experiments in interdisciplinarity from this perspective means exploring openly and collaboratively how research practices and how social, political, and economic dimensions interplay around and within a specific research project.

## Abbreviations

EC: European Commission
ICT: Information Communication Technology
iGEM: International Genetically Engineered Machine
PI: principal investigator
R&D: research and development
RRI: responsible research and innovation
SSH: social sciences and humanities
STS: Science and Technology Studies
UV: University of Valencia
